# Dietary Inorganic Nitrate Protects Hepatic Ischemia-Reperfusion Injury Through NRF2-Mediated Antioxidative Stress

**DOI:** 10.3389/fphar.2021.634115

**Published:** 2021-06-07

**Authors:** Shaorong Li, Hua Jin, Guangyong Sun, Chunmei Zhang, Jinsong Wang, Hufeng Xu, Dong Zhang, Songlin Wang

**Affiliations:** ^1^ Salivary Gland Disease Center and Beijing Key Laboratory of Tooth Regeneration and Function Reconstruction, Beijing Laboratory of Oral Health, Capital Medical University School of Stomatology, Beijing, China; ^2^ Immunology Research Center for Oral and Systemic Health, Beijing Friendship Hospital, Capital Medical University, Beijing, China; ^3^ Beijing Key Laboratory of Tolerance Induction and Organ Protection in Transplantation, Beijing, China; ^4^ Department of Biochemistry and Molecular Biology, Capital Medical University School of Basic Medicine, Beijing, China

**Keywords:** hepatic ischemia reperfusion injury (HIRI), nitrate, oxidative stress, NRF2, oral administration

## Abstract

**Objectives:** Hepatic ischemia-reperfusion injury (HIRI) is of common occurrence during liver surgery and liver transplantation and may cause hepatic impairment, resulting in acute liver dysfunction. Nitrate plays an important physiological regulatory role in the human body. Whether dietary nitrate could prevent HIRI is, however, unknown.

**Methods:** A HIRI mouse model was established in that the blood supply to the median lobe and left lateral lobe was blocked for 60 min through the portal vein and related structures using an atraumatic clip. Sodium nitrate (4 mM) was administrated in advance through drinking water to compare the influence of sodium nitrate and normal water on HIRI.

**Results:** Liver necrosis and injury aggravated after HIRI. The group treated with sodium nitrate showed the lowest activities of plasma aminotransferase and lactate dehydrogenase and improved outcomes in histological investigation and TUNEL assay. Mechanistically, sodium nitrate intake increased plasma and liver nitric oxide levels, upregulated nuclear factor erythroid 2-related factor 2 (NRF2)–related molecules to reduce malondialdehyde level, and increased the activities of antioxidant enzymes to modulate hepatic oxidative stress.

**Conclusions:** Dietary inorganic nitrate could prevent HIRI, possibly by activating the NRF2 pathway and modulating oxidative stress. Our study provides a novel therapeutic compound that could potentially prevent HIRI during liver transplantation or hepatic surgery.

## Introduction

Hepatic ischemia-reperfusion injury (HIRI) is common during liver resection or transplantation, which remains a major cause of hepatic failure following hepatic surgery (Mendes-Braz et al., 2012). A series of pathological alterations are referred to liver ischemia-reperfusion (IR) injury, including initial sterile hypoxic or ischemic tissue injury, reperfusion-induced oxidative stress, inflammatory response, and microvascular dysfunction (Massip-Salcedo et al., 2007), and effective prevention or treatment methods are still lacking in clinics.

The mainstream research direction includes oxidative stress, immune response, and inflammatory response (Dar et al., 2019). Nitric oxide (NO) plays an important role in organ IR injuries (Peralta et al., 2013). Low tissue oxygen tension significantly decreased the oxygen-dependent NO synthesis of endothelial NO synthase during ischemia (Webb et al., 2004). Furthermore, overproduction of malondialdehyde (MDA) in the reperfusion phase further consumes endogenous NO (Weitzberg et al., 2010). Reduced bioavailability of NO can lead to endothelial and microvascular functional imbalance, bringing about the “no-reflow phenomenon” after ischemic tissue reperfusion is initiated (Eltzschig and Eckle, 2011). Therefore, safe and effective methods to reduce oxidative stress, alleviate inflammation, and maintain NO bioavailability may become a new strategy for the prevention and treatment of hepatic IR injury.

Nitrates are quite abundant in our ordinary daily diets, especially in green leafy vegetables such as spinach, lettuce, or beetroot (Song et al., 2015). In oral microenvironment, commensal nitrate-reducing bacteria effectively degrade nitrate to nitrite, which is swallowed with 1 L saliva per day and continuously enters the circulation (Mensinga et al., 2003; Lundberg et al., 2011). Nitrate can be metabolized *in vivo* to form NO and other bioactive nitrogen oxides (Lundberg et al., 2008). In contrast to NO synthases, the nitrate–nitrite NO pathway is independent of oxygen and L-arginine. Therefore, the formation of NO from this source is unaffected during IR damage (Zweier et al., 1995). The protective response of nitrite on IR damage have been verified in multiple organs or tissues such as the brain (Jung et al., 2006), lungs (Sugimoto et al., 2012), and heart (Omar et al., 2016; Liu et al., 2020). However, the potential therapeutic value of inorganic nitrate in liver IR injury remains controversial.

In this study, we applied a mouse HIRI model to investigate the hepatic and systemic protection of dietary inorganic nitrate supplementation and explore the underlying mechanism of action.

## Materials and Methods

### Mice

This study was approved by the Institutional Animal Care and Ethics Committee at Beijing Friendship Hospital (IACUC ID: 18-2009) and performed in accordance with the Ethical Guidelines for Animal Studies.

Male C57BL/6 mice (8-week-old) weighing 20–30 g were obtained from Beijing Vital River Laboratory (Beijing, China) and housed under a specific pathogen-free, temperature-controlled, and humidity-controlled environment with free access to rodent chow and tap water at the animal facilities at Beijing Friendship Hospital (Beijing, China).

### Reagents and Antibodies

Sodium nitrate was obtained from Sigma–Aldrich (S5506 Louis, MO, United States). Hydrogen peroxide (H_2_O_2_, 344945, LiErkang, Shandong, China), sodium nitroprusside dihydrate (SNP, S0015, Beyotime, Shanghai, China), ML385 (A NRF2 inhibitor, S8790, Selleck, CA, United States), and reactive oxygen species (ROS) assay kit (50101ES01, YEASEN, Shanghai, China) were purchased from the relative company. Anti-NRF2 (12721, Cell Signaling Technology, MA, United States), anti-KEAP1(8047S, Cell Signaling Technology, MA, United States), anti-Bcl-2 (3498T, Cell Signaling Technology, MA, United States), anti-Bcl-xL (2764T, Cell Signaling Technology, MA, United States), anti-GAPDH (5174T, Cell Signaling Technology, MA, United States) and anti-HO1 (43966S, Cell Signaling Technology, MA, United States) was provided by Cell Signaling Technology, and histone-H3 (17168-1-AP, ProteinTech, IL, United States) was procured from ProteinTech. Anti-NQO1 (ab34173, Abcam, MA, United States) and anti-Ly6G (ab122501, Abcam, MA, United States) was bought from Abcam. APC-Annexin V (640920, BioLegend, CA, United States) was purchased from BioLegend.

### Mouse HIRI Model

A mouse HIRI model was successfully established as previously described (Jin et al., 2019). In brief, mice were anesthetized and heparinized to prevent blood clotting. A midline laparotomy incision was performed and an atraumatic clip was used to completely clamp the hepatic artery and portal vein, causing ischemia of the left lateral and median lobes of the liver. The mice were wrapped with a heating pad to maintain body temperature at 37°C. After 60 min, the atraumatic clip was removed to allow reperfusion. Then the mice were sutured. 6 h later, the liver and plasma were collected for measurement (Abe et al., 2009).

### Plasma Transaminase Activities and Lactate Dehydrogenase (LDH) Enzyme Activities

Plasma alanine aminotransferase (ALT) and aspartate aminotransferase (AST) activities were determined as quantitative indices of liver injury using an alanine aminotransferase assay kit (C009-2, NJJC Bio Inc., Nanjing, China) and aspartate aminotransferase assay kit (C010-2, NJJC Bio Inc., Nanjing, China), respectively, according to the manufacturer’s instructions. Plasma LDH activity was analyzed as per the method recommended for the lactate dehydrogenase assay kit (A020-2, NJJC Bio Inc., Nanjing, China).

### Nitrate, Nitrite, and NO Levels in the Plasma and Liver Tissue

Plasma and liver tissue were obtained and homogenized to collect the supernatant. Before the assay, samples were filtered using 10,000 MW filters and diluted. The total nitric oxide and nitrate/nitrite parameter assay kit (KGE001, R&D, MN, United States) was employed to determine the concentration of nitrate and nitrite, while a mouse Nitric Oxide (NO) ELISA Kit (EK18797, SAB, Maryland, United States) was used to measure the NO level.

### Histological and Immunohistochemical Analysis

Liver samples were fixed in phosphate-buffered formalin and embedded in paraffin. Sections (5 µm) were stained with hematoxylin and eosin. The stained sections were semiquantitatively evaluated and examined in a blinded manner for each liver sample and scored from 0 to 4 to assess the condition of liver necrosis and steatosis, sinusoidal congestion, and hepatic cytoplasmic vacuolation-infiltrating polymorphonuclear leukocytes according to Suzuki Score (Suzuki et al., 1993).

For immunohistochemical analysis, neutrophils were evidenced in paraffin sections using anti-Ly6G antibody. Ly6G positive cells were calculated by Image J software (NIH, MD, United States).

### Terminal Deoxynucleotidyl Transferase dUTP Nick End Labeling (TUNEL) Assay

Paraffinized samples were sectioned at 6 μm thickness, mounted on silane-coated glass slides. TUNEL staining was performed with a Click-iT^®^ Plus TUNEL Assay Kit (C10617, Thermo Fisher Scientific Inc., PA, United States) according to the manufacturer’s instructions. Images were captured under a confocal microscope and the number of TUNEL-positive cells was calculated using Image J software (NIH, MD, United States).

### Antioxidant Enzyme Activities and MDA Level of the Liver

Catalase (CAT) activity in the liver was determined using a catalase (A007-1, NJJC Bio Inc., Nanjing, China) assay kit. Glutathione peroxidase (GSH-PX) and superoxide dismutase (SOD) activities were measured using glutathione peroxidase (A005-1, NJJC Bio Inc., Nanjing, China) and superoxide dismutase (A001-3, NJJC Bio Inc., Nanjing, China) assay kits, respectively. MDA level was detected using an MDA assay kit (A003-1, NJJC Bio Inc., Nanjing, China).

### Determination of Plasma Cytokine Levels

According to the manufacturer's instructions, the multiple cytokine levels of plasma were measured using the LEGENDplex™ mouse inflammation panel (740150, BioLegend, CA, United States). Then, the prepared samples were tested using Aria II flow cytometer (BD Biosciences), and the data were analyzed by LEGEND plex software v8.0 (Biolegend, CA, United States).

### Cell Culture

Mouse normal hepatocytes (AML12 cells) were purchased from the American Type Culture Collection (VA, United States). The culture media includes 1:1 mixture of DMEM and F12-K medium plus 1% 100 × Insulin–Transferrin–Selenium–Ethanolamine (51500056, Thermo Fisher Scientific Inc., PA, United States), 50 U/ml penicillin, and 50 μg/ml streptomycin at 37°C under 5% CO_2_.

Approximately 1×10^5^ cells per well were cultured in a 24-well plate for 12 h. 500 μM H_2_O_2_ was used to mimic the oxidative stress environment. Exogenous NO and hepatocyte NRF2 inhibition were provided by 25 μM SNP and 4 μM ML385, respectively.

### Intracellular NO Levels

An intracellular NO Detected Kit (S0021S, Beyotime, Shanghai, China) was used to test the level of NO. According to the instructions, the intracellular NO level was measured and analyzed.

### Flow Cytometry Analysis of ROS and Apoptosis

Cells were suspended and centrifuged. Part of the centrifuged cells were rinsed with PBS and incubated with ROS probe. Other cells were rinsed with Annexin V binding buffer and incubated with Annexin V. After the process finished, all samples were acquired on a FACS Aria II flow cytometer (BD Biosciences), and data were analyzed using FlowJo software (Tree Star, OR, United States).

### Real-Time Polymerase Chain Reaction (RT-PCR)

Total RNA was extracted from the liver tissue using an Eastep Super Total RNA Extraction Kit (LS1040, Shanghai Promega, Shanghai, China) in accordance with the manufacturer’s protocol and reverse-transcribed to cDNA using a PrimeScript RT Reagent Kit (RR037A, TaKaRa, Tokyo, Japan). Quantitative RT-PCR analysis was performed using an ABI 7500 Sequence Detection System (Applied Biosystems, CA, United States). The PCR mixture comprised 10 μl SYBR Green Master Mix, 0.5 μM forward and reverse primers, and 1 μl cDNA sample. After normalization of target gene expression, the data was quantified by the 2^−ΔΔCt^ method. The genes and primer sequences are listed in Supplementary Table S1.

### Western Blot Analysis

The liver tissues were weighed and prepared by manual grinding on ice. Total protein was used RIPA (Solarbio, Beijing, China) while nuclear and cytosolic proteins were extracted using a nuclear and cytosolic extraction reagent kit (P1200-100, Applygen, Beijing, China). The protein concentration was determined using a bicinchoninic acid protein assay, as recommended by the manufacturer. Equal amounts (40 μg) of proteins were subjected to 10% sodium dodecyl sulfate polyacrylamide gel electrophoresis (SDS-PAGE) and transferred onto polyvinylidene fluoride (PVDF) membranes (10600023, GE, MA, United States). The membranes were incubated with primary antibodies against NRF2 (1:1000 dilution) and histone-H3 (1:1000 dilution), KEAP1 (1:1000), NQO1 (1:1000), HO1 (1:1000), Bcl-2 (1:1000), Bcl-xL (1:1000), GAPDH (1:2000), and then probed with secondary antibodies conjugated to HRP (ZB-2301, Zhongshan Golden Bridge Biotechnology Co., Beijing, China). Relative expression was analyzed by Image J software (NIH, MD, United States).

### Statistical Analysis

Sample collection and data analysis were performed by different members according to the double-blind principle. The data were analyzed with GraphPad Prism 8.0 software (GraphPad Software, CA, United States). Results are presented as mean ± standard error of mean (SEM). Student’s t-test was used to compare difference between two groups with normal distribution or Kruskal–Wallis test for others. Among multiple groups, one-way ANOVA with post-hoc test for the normal distribution and Kruskal–Wallis test for others. *p < 0.05, **p < 0.01, and ***p < 0.001 were considered significant, and *NS* was not significant. Statistical analysis was performed using SPSS 20.0.

## Results

### Dietary Nitrate Attenuated IR-Induced Liver Injury

**FIGURE 1:**
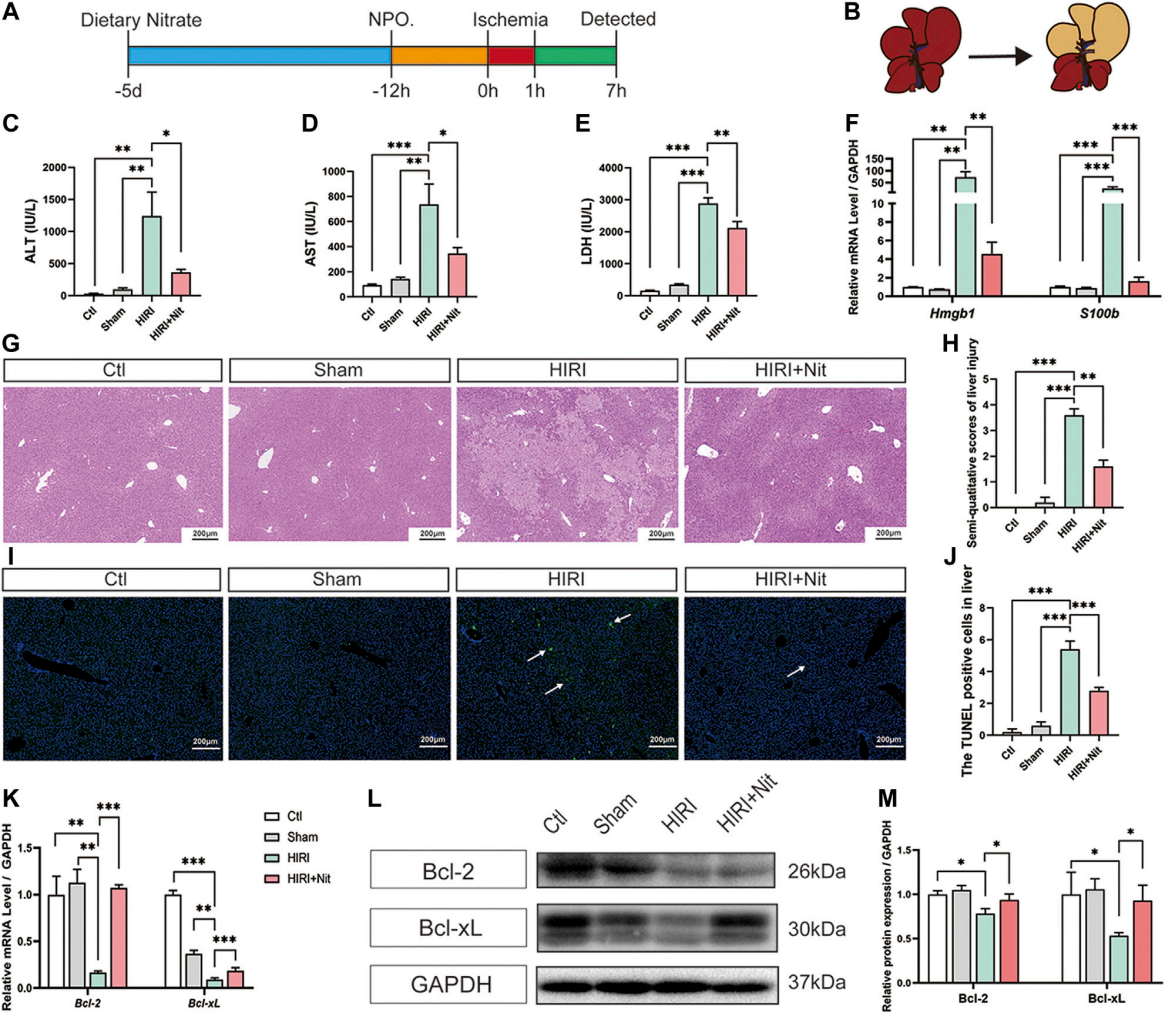
Dietary nitrate prevented hepatic ischemia-reperfusion injury in mice. **(A)** Model of hepatic ischemia in which dietary nitrate was administered 5 days before operation (*n* = 5/group). **(B)** A ventral view of clamping the portal vein and hepatic artery to induce ischemia in the left lateral and median lobes of the liver. plasma activities of **(C)** ALT, **(D)** AST, and **(E)** LDH. **(F)** Relative mRNA expression of *Hmgb1* and *S100b*. **(G)** Histological changes (hematoxylin and eosin staining) of Ctl, Sham, HIRI, and HIRI + Nit groups at 100 × magnification. **(H)** Semiquantitative score of histological study. **(I)** TUNEL assay of the Ctl, Sham, HIRI, and HIRI + Nit groups at 100 × magnification. **(J)** Quantitative score of TUNEL assay. **(K)** Relative mRNA levels of *Bcl-2* and *Bcl-xL.* **(L)** Western blot analysis of Bcl-2 and Bcl-xL and **(M)** statistical analyses. Data are expressed as mean ± SEM, *p<0.05, **p<0.01, ***p<0.001, and *NS* denotes no significance.

The HIRI model was established as shown in Figures 1A,B. Mice were administrated with 4 mM dietary nitrate through drinking water 5 days before the operation. The water intake, food intake, and body weight of each mouse were detected (Supplementary Figure S1), while no difference was found among the groups. The total intake dosage of sodium nitrate was 78.59 ± 6.93 μg/day per kilogram of weight.

As shown in Figures 1C–E, plasma ALT, AST, and LDH activities were significantly higher in HIRI group than in the control group and sham group. Mice exposed to 4 mM nitrate for 5 days prior to HIRI showed a significant decrease in the activities of ALT, AST, and LDH as compared with unpretreated mice.

To investigate other IR-related factors, we also detected the damage-associated molecular pattern molecules (DAMPs) and revealed that the mRNA level of *Hmgb1* and *S100b* was significantly increased in HIRI group and remarkably decreased in nitrate intake group (Figure 1F).

Consistent with the changes in plasma markers, morphological alterations such as tubular necrosis and infiltration of inflammatory cells were obvious in the HIRI liver (Figure 1G), while nitrate pretreatment had the ability to attenuate IR-induced liver damage. The histopathological score of the liver also revealed the nitrate-mediated significant amelioration of IR-related liver injury (Figure 1H).

TUNEL assay (Figures 1I,J) showed that apoptosis level increased after IR but significantly decreased by nitrate pretreatment. RT-PCR of liver tissues (Figure 1K) revealed the downregulation in the expression of the anti-apoptosis genes *Bcl-2* and *Bcl-xL* in HIRI group, and an upregulation in 4 mM nitrate-treated group. The protein expression (Figures 1L,M) also showed the similar consequences that the lowest expression level in HIRI mice and a reversion could be seen in nitrate intake mice. The expression of anti-apoptosis gene and protein was consistent with the result of TUNEL assay.

### Dietary Nitrate Reduced IR-Associated Inflammatory Responses

**FIGURE 2:**
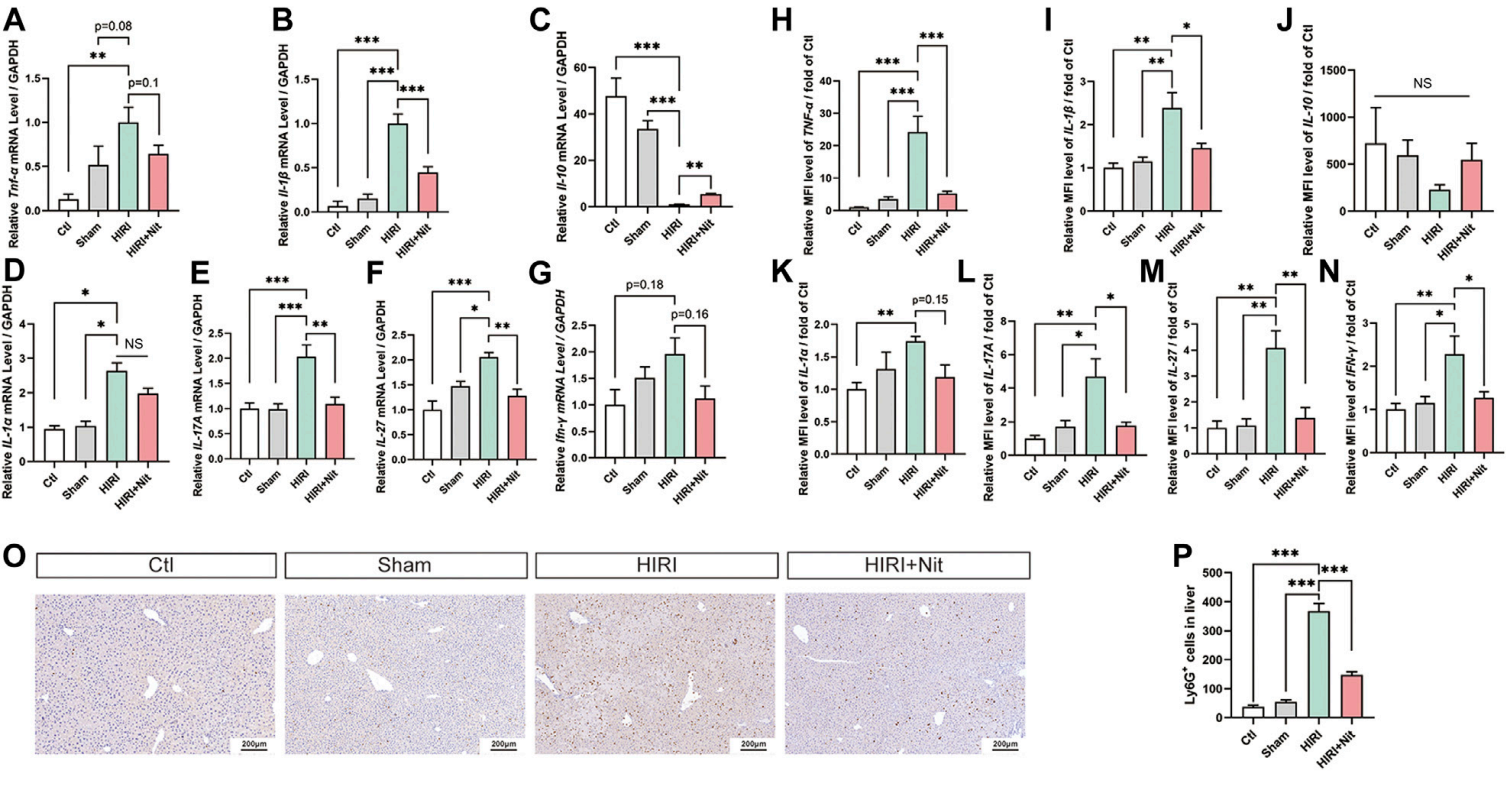
Dietary nitrate reduced IR-associated inflammatory responses in mice. **(A)** Relative mRNA level of *Tnf-*α, **(B)** *Il-1*β, **(C)** *Il-10,* **(D)** *Il-1*α, **(E)** *Il-17A*, **(F)** *Il-27*, and **(G)** *Ifn-γ*. **(H)** Plasma levels of TNF-α, **(I)** IL-1β, **(J)** IL-10, **(K)** IL-1α, **(L)** IL-17 and **(M)** IL-27, and **(N)** IFN-*γ*. **(O)** Immunohistology of Ly6G in Ctl, Sham, HIRI, and HIRI + Nit groups at 100 × magnification. **(P)** Quantitative analysis of Ly6G-positive cells. Data are expressed as mean ± SEM, *p<0.05, **p<0.01, ***p<0.001, and *NS* denotes no significance.

Tumor necrosis factor (TNF)-α and interleukin (IL)-1β are considered as proinflammatory factors, while IL-10 is an anti-inflammatory cytokine. As shown in Figures 2A–G, the relative mRNA levels of *Tnf-*α and *Il-1*β increased and that of *Il-10* decreased in HIRI group as compared to those in control groups. In addition, the levels of *Il-1α*, *Il-17A*, *Il-27*, and *interferon (Ifn)*-*γ* also increased in HIRI group*.* However, nitrate administration downregulated the expression of *Tnf-*α, *Il-1*β*, Il-1*α, *Il-17A*, *Il-27,* and *Ifn-γ* in the liver tissue. *Il-10* expression was the lowest in HIRI group but was significantly restored in 4 mM nitrate pretreatment group.

We also evaluated the changes in the plasma levels of several pro-inflammatory factors, including TNF-α, IL-1β, IL-10, IL-1α, IL-17A, IL-27, and IFN-*γ.* As shown in Figures 2H–N, the plasma levels of above cytokines were altered, consistent with the results of RT-PCR. The levels of TNF-α, IL-1β, IL-1α, IL-17A, IL-27, and IFN-*γ* were higher in HIRI group while IL-10 was lower than in controls. However, nitrate administration in HIRI + Nit group decreased TNF-α, IL-1β, IL-1α, IL-17A, IL-27, and IFN-*γ* levels in the plasma while IL-10 increased as compared to unpretreated HIRI group.

In IR model, the augment of neutrophils could be seen remarkably. To investigate the immune and inflammatory condition, the infiltration of neutrophils in the liver was tested by immunohistochemistry. Nitrate intake markedly decreased Ly6G positive cells in liver with HIRI (Figures 2O,P).

### Dietary Nitrate Increased Nitrate, Nitrite and NO in the Plasma and Liver and Reduced IR-Associated Oxidative Stress

**FIGURE 3:**
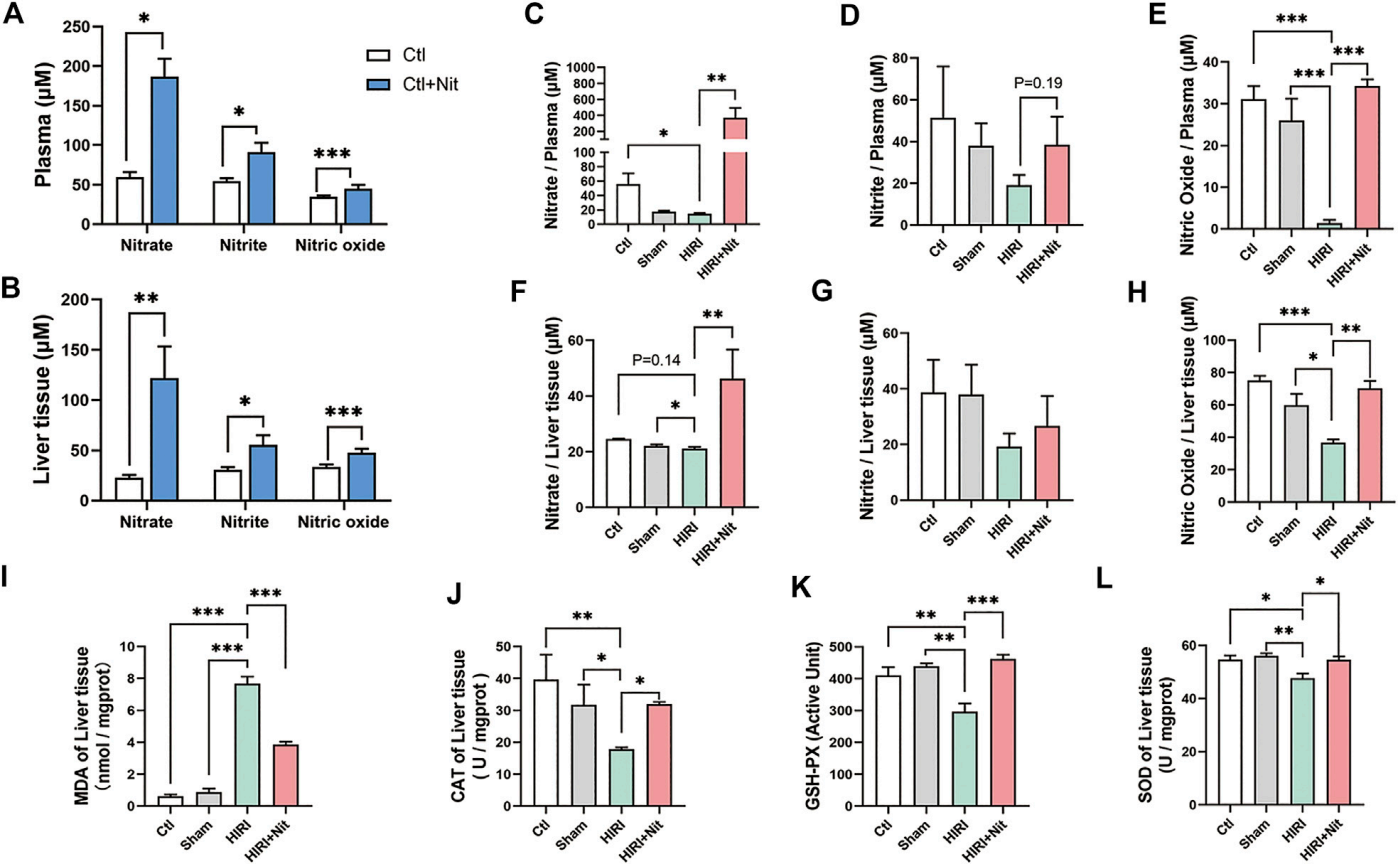
Effects of 4 mM dietary nitrate on levels of nitrate, nitrite, and NO as well as the oxidative index in the liver tissue. **(A)**The nitrate, nitrite, and NO levels of the plasma in Ctl and Ctl + Nit groups. **(B)**The nitrate, nitrite, and NO levels of the liver tissue in Ctl and Ctl + Nit groups. The levels of **(C)** nitrate, **(D)** nitrite, and **(E)** NO in the plasma of Ctl, Sham, HIRI, and HIRI + Nit groups. The levels of **(F)** nitrate, **(G)** nitrite, and **(H)** NO in the liver tissue of Ctl, Sham, HIRI, and HIRI + Nit groups. **(I)** The levels of MDA and the activities of **(J)** CAT, **(K)** GSH-PX, and **(L)** SOD in Ctl, Sham, HIRI, and HIRI + Nit groups. Data are expressed as mean ± SEM, *p<0.05, **p<0.01, ***p<0.001, and *NS* denotes no significance.

To explore the functional role of dietary nitrate in HIRI, we measured the concentrations of nitrate, nitrite, and NO in the plasma and liver tissues. As shown in Figures 3A,B, 5 days of 4 mM nitrate intake could significantly increase nitrate, nitrite, and NO levels both in plasma and liver of normal mice. Without nitrate administration, the nitrate levels in plasma were lower in HIRI group. The mice pretreated with 4 mM nitrate showed statistically higher levels of plasma nitrate than mice from HIRI group (Figure 3C). The plasma nitrite levels in the liver also revealed the similar tendency although without significant difference (Figure 3D).

As a key functional molecule indicative of nitrate activity, we observed that the NO levels in the plasma of HIRI group were significantly lower than those in the liver of the control group and the sham group (Figure 3E). However, the mice treated with nitrate showed statistically higher levels of liver NO than the mice from HIRI group. Similar changes in liver tissue could be seen in Figures 3F–H. Dietary nitrate could reverse the reduction of nitrate, nitrite, and NO in liver tissue.

The level of MDA in the liver (Figure 3I), an index of lipid peroxidation, was significantly higher in HIRI group than that in the control and sham group, but 4 mM nitrate administration significantly alleviated the levels of MDA.

As shown in Figures 3J–L, the activities of antioxidant enzyme CAT, GSH-PX, and SOD were significantly lower in HIRI group than those in the control and sham group, possibly owing to the depletion of the antioxidant pool that was consumed to remove excess of ROS produced during HIRI. The antioxidant enzyme activities were restored in mice administered with 4 mM nitrate.

### Dietary Nitrate Activated the NRF2 Pathway in HIRI

**FIGURE 4:**
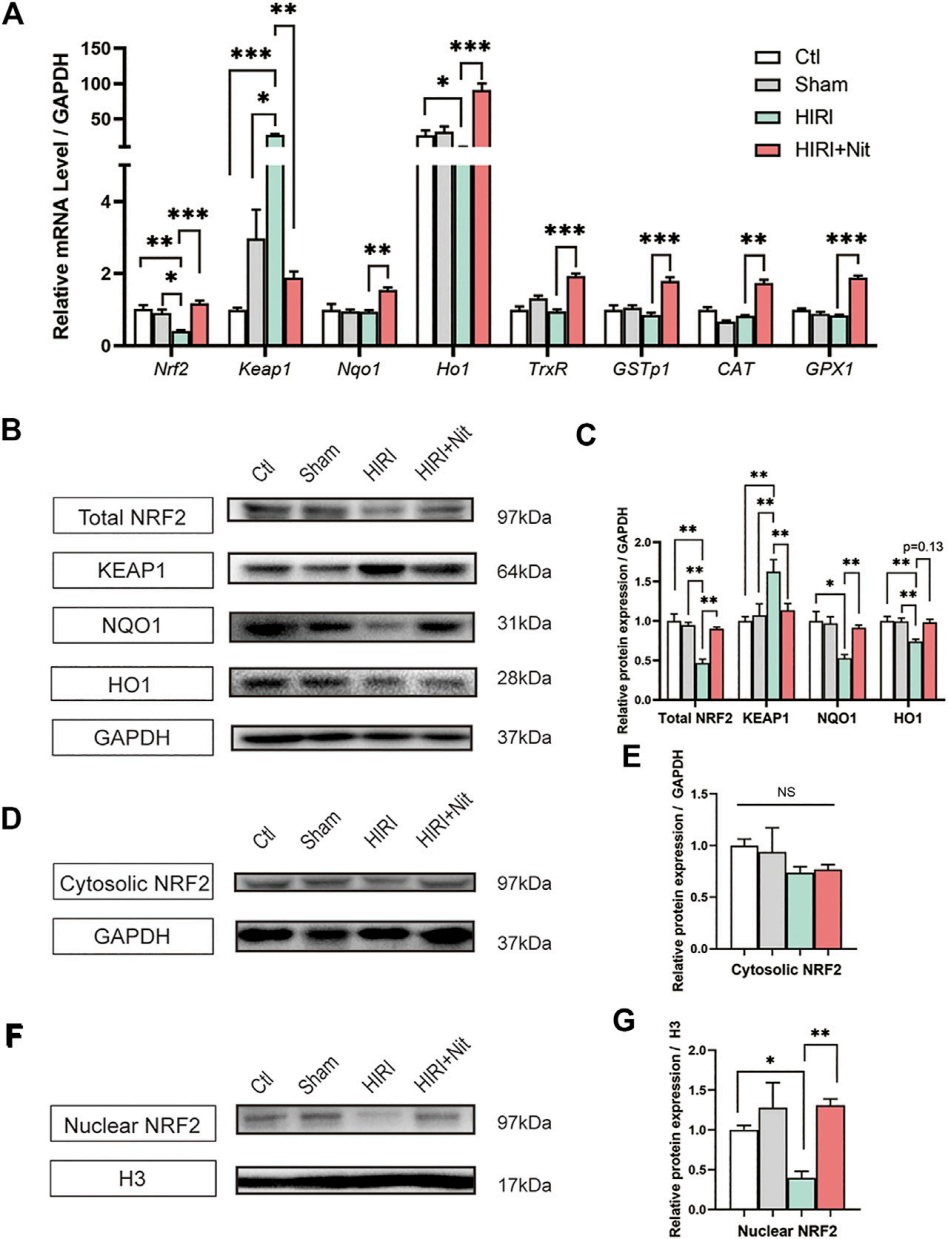
Effects of nitrate pretreatment on NRF2 activation in ischemia-induced mice. **(A)** Relative mRNA level of *Nrf2, Keap1, Nqo1, Ho1, TrxR, GSTp1, CAT, GPX1*. **(B)** Western blot analysis of total NRF2, KEAP1, NQO1, HO1 and **(C)** statistical analyses. **(D)** Western blot analysis of cytosolic NRF2 and **(E)** statistical analyses. **(F)** Western blot analysis of nuclear NRF2 and **(G)** statistical analyses. Data are expressed as mean ± SEM, *p<0.05, **p<0.01, ***p<0.001, and *NS* denotes no significance*.*

Previous reports have shown that NRF2 activation is associated with oxidative stress and exerts a strong protective effect against hepatotoxicity through increased autophagy (Bellezza et al., 2018). To explore the potential protective mechanisms of nitrate on liver, we examined the NRF2 pathway. Our findings (Figure 4A) indicated that nitrate pretreatment efficiently increased the mRNA levels of *Nrf2* and *Nrf2*-related genes such as quinone oxidoreductase 1 (*Nqo1*), heme oxygenase 1 (*Ho1*), thioredoxin reductase (*TrxR*), *CAT*, and Glutathione Peroxidase 1 (*GPX1*). Further, nitrate pretreatment downregulated the expression of NRF2 repressor protein Kelch-like ECH-associated protein 1 (*Keap1*). Also, relative protein expression revealed that NRF2, NQO1, and HO1 in total protein (Figures 4B,C) reduced in HIRI group than those in control and sham group, while dietary nitrate could increase the expression significantly. Contrast to NRF2, the protein expression of KEAP1 was increased in HIRI group and decreased in HIRI + Nit group. The results of total protein expression were consistent with the mRNA levels. When NRF2 activated, parts of it should transport into the nucleus to exert function, so we also detected the expression of cytosolic and nuclear NRF2. We found that the expression had no significance in cytosolic NRF2 (Figures 4D,E), however, remarkably alteration of NRF2 was revealed in nucleus (Figures 4F,G). A significant increase could be observed in mice treated with nitrate.

### NO Pretreatment Attenuated Oxidative Stress Induced by H_2_O_2_ and Upregulated NRF2 *in vitro*

**FIGURE 5:**
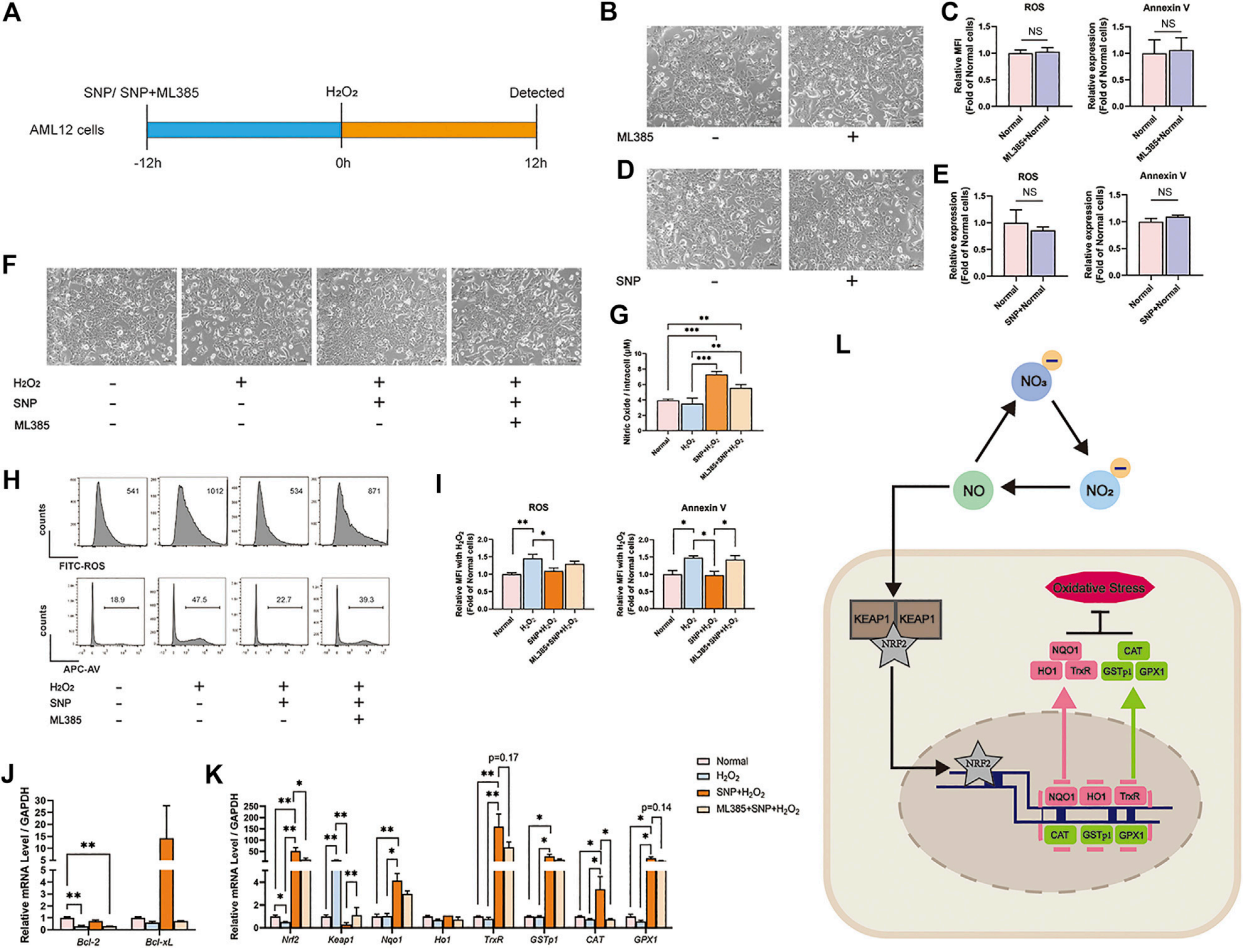
SNP (NO donor) pretreatment attenuated oxidative stress induced by H_2_O_2_ and upregulated NRF2 *in vitro*, while ML385 (NRF2 inhibitor) reversed the protective effect of SNP on H_2_O_2_-stimulated AML12 cells. **(A)** SNP or SNP + ML385 was administered 12 h before the 12 h stimulation of H_2_O_2_. **(B)** Morphology of AML12 cells in Normal and Normal + ML385 groups. **(C)** Relative MFI of ROS, Annexin V, in Normal and Normal + ML385 groups. **(D)** Morphology of AML12 cells in Normal and Normal + SNP groups. **(E)** Relative MFI of ROS, Annexin V, in Normal and Normal + SNP groups. **(F)** Morphology of AML12 cells in Normal, H_2_O_2_, SNP + H_2_O_2_, and ML385 + SNP + H_2_O_2_ groups. **(G)** The level of NO in Normal, H_2_O_2_, SNP + H_2_O_2_, and ML385 + SNP + H_2_O_2_ groups. **(H)** Representative flow cytometric results of ROS, Annexin V, and **(I)** statistical analyses. **(J)** Relative mRNA level of *Bcl-2* and *Bcl-xL.* **(K)** Relative mRNA level of *Nrf2, Keap1, Nqo1, Ho1, TrxR, GSTp1, CAT,* and *GPX1* in Normal, H_2_O_2_, SNP + H_2_O_2_, and ML385 + SNP + H_2_O_2_ groups. **(L)** Mechanisms by which dietary nitrate protects HIRI. Dietary nitrate is converted to NO, which is transported through the cell membrane and disintegrates KEAP1 and NRF2. NRF2 is transferred into the nucleus, wherein it activates downstream genes and transcribes related proteins to modulate oxidative stress. Data are expressed as mean ± SEM, *p<0.05, **p<0.01, ***p<0.001, and *NS* denotes no significance.

To validate the mechanism of NRF2-pathway, AML12 cells were treated with 500 μM H_2_O_2_ to mimic the oxidative condition *in vitro*. SNP, a common NO donor, provided exogenous NO in the culture medium. A NRF2 inhibitor ML385 (Singh et al., 2016) was also used in this study to block NRF2-pathway in AML12 cells. As shown in Figure 5A, AML12 cells were pretreated with SNP or SNP + ML385 for 12h, and followed by 12h-H_2_O_2_ stimulation. AML12 cells treated with 4 μM ML385 alone for 24 h had no changes of morphology (Figure 5B), ROS, and Annexin V levels (Figure 5C). Also, AML12 cells treated with 25 μM SNP alone for 24 h had no alterations of morphology (Figure 5D), ROS, and Annexin V levels (Figure 5E). AML12 cells showed (Figure 5F) shrinkage, uncleared outline and increased intercellular particles after H_2_O_2_ loading. SNP pretreatment could maintain the normal morphology of AML12 cells, however, the administration with ML385 could reverse the protection of SNP on AML 12 cells. As shown in Figure 5G, although the intracellular concentrations of NO were increased in both SNP-treated cells, the protection of AML12 cells toward oxidative stress induced by H_2_O_2_ could only be seen in SNP pretreated cells but not in SNP and ML385 combination treated cells (Figures 5H–J). Furthermore, the relative mRNA levels of *Nrf-2, Nqo1,* and associated genes were lower and *Keap1* was higher in H_2_O_2_ stimulated cells, SNP pretreatment restored the changes accordingly. However, blocking the NRF2 pathway in AML12 cells by ML385 could partially inhibit the alteration induced by SNP (Figure 5K). Taken together, NO pretreatment attenuated oxidative stress induced by H_2_O_2_ mainly through NRF2 pathway.

## Discussion

Nitrate is thought to be harmful owing to the potential production of carcinogenic nitrosamines (Bedale et al., 2016). However, there is no clear evidence regarding dietary nitrate-mediated increase in the occurrence of cancer (Bryan et al., 2012); instead, studies have highlighted the potential benefits of nitrate. For years, we have performed several studies on the management of oral and systemic diseases using nitrate, and demonstrated that nitrate can effectively prevent Sjogren's syndrome (Xia et al., 2015), promote the effect of chemoradiotherapy (Chang et al., 2019), prevent gastrointestinal stress (Jin et al., 2013), facilitate weight loss (Ma et al., 2020), and alleviate aging (Wang et al., 2018).

Systemic nitrates circulate in the blood, saliva, and tissues and are absorbed after a nitrate-rich diet with a peak plasma level of 15–30 min with a half-life period of about 5–8 h (Witter et al., 1979; Lundberg et al., 2011). Due to the bioavailability of nitrate in the stomach and small intestine, it is almost completely absorbed, with about 75% excreted in the urine and the rest reabsorbed by the kidneys, biliary tract, and salivary glands (Kahn et al., 1975; Fritsch et al., 1985). Normally, up to 25% recycled nitrate can be found in salivary glands, and nitrate concentrations in salivary glands are 10 times higher than in plasma (Spiegelhalder et al., 1976) . The role of nitrate in the human body mainly depends on the nitrate–nitrite–NO axis, and is mediated through NO (Witter et al., 1979). In this process, nitrate is converted to nitrite by oral and gastrointestinal bacteria. Nitrite is extremely unstable and gets converted to NO through enzymatic reactions (Bryan and Ivy, 2015).

The results of this study showed that oral intake of inorganic nitrate could effectively prevent HIRI. Previous studies have revealed the preventive effects of intravenous nitrite on HIRI; however, nitrite is unstable and inconvenient for common use (Shiva et al., 2007). Further, intravenously administrated nitrate may not perform the necessary functions (Duranski et al., 2005). The differences in results may be attributed to the different intake method adopted in our study. Oral and gastrointestinal bacteria are of the utmost importance for nitrate transfer to NO (Gaston et al., 1994). However, the access of intravenously injected nitrate to bacteria was limited, which may prevent the conversion of nitrate to nitrite and then into NO.

There were lots of indices about oxidative stress, including ROS, antioxidant enzymes, and MDA. ROS were highly reactive and attack biomolecules including proteins, DNA, and lipids such as polyunsaturated fatty acids. This phenomenon was generally known as “oxidative stress”. The polyunsaturated fatty acid arachidonic acid could be peroxidized to finally form MDA. This particular reaction of ROS with lipids was generally known as “lipid peroxidation” (Tsikas, 2017). Previous study showed that a time-dependent increase in lipid peroxidation products was observed during ischemia in a model of HIRI which indicated MDA was a key factor of oxidative stress process in HIRI (Fukai et al., 2005). Our study revealed that dietary inorganic nitrate could regulate the level of oxidative stress, which is consistent with the results of previous studies. A previous study evaluated the role of dietary nitrate in unilateral renal IR injury and found that nitrate could regulate the level of oxidative stress (Yang et al., 2017). However, the mechanism of action of nitrate on oxidative stress was not clarified. Similar conclusions have been derived by intravenous nitrite administration in heart IR injury (Shiva et al., 2007).

NRF2 is a member of the cap ‘n’ collar family of basic region leucine zipper transcription factors that was first identified, cloned, and characterized in 1994 (Moi et al., 1994). Growing evidence implies that the activation of NRF2 signaling and the upregulation of downstream antioxidant enzymes are crucial to suppress oxidative stress and maintain cellular homeostasis (Bellezza et al., 2018). Many drugs exert anti-cancer, anti-apoptosis, anti-oxidation, and anti-inflammatory effects by regulating the expression and activity of NRF2 (Copple et al., 2010; Sun et al., 2015). Also, a research about kidney IR showed that NRF2-knockout mice had a worse performance compared to wild type mice (Liu et al., 2009). In this study, we focused on NRF2 to see whether it could modulate hepatic injury caused by IR.

On NRF2 pathway, several key genes take vital roles. KEAP1, a NRF2 repressor and the main intracellular regulator of NRF2, has five domains, each of which is important for inhibiting NRF2 activity (Bellezza et al., 2018). NQO1 is a cytosolic flavin that catalyzes the two-electron reduction and detoxification of quinones and other exogenous and endogenous chemicals in the redox cycle (Vasiliou et al., 2006). HO1 is a cytoprotective endogenous enzyme that exhibits both anti-inflammatory and anti-oxidative effects by catalyzing the first and rate-limiting step (Klaassen and Reisman, 2010). We investigated the expression of KEAP1, NQO1, and HO1 and found that total protein expression of NRF2 and NQO1 were increased under dietary nitrate. Importantly, we found nuclear NRF2 also upregulated compared to the HIRI group which means NRF2 had nuclear translocation to further influence the downstream genes.

In order to verify the *in vivo* results, we introduced *in vitro* study. With H_2_O_2_ stimulation, the levels of ROS and Annexin V of AML12 cells increased significantly, while SNP, which could release NO, could decrease the oxidative stress and apoptosis caused by H_2_O_2_. Also, during SNP loading, NRF2 pathway was activated. In order to ensure the function of NRF2, we employed a NRF2 inhibitor-ML385, under a concentration without affecting the normal cells, could inhibit the expression of NRF2 associated genes. And more importantly, NRF2 inhibition could reverse the protective effects of SNP on H_2_O_2_ treated AML12 cells. By experimental verification, we hypothesized that NO, which was derived from nitrate, could be transported through the cell membrane, and activate the NRF2 pathway. More NRF2 protein is transported into the nucleus to mediate the transcription of related genes. Finally, the proteins that were transcribed by these genes play pivotal roles in modulating hepatic oxidative stress (Figure 5L).

In summary, here we found that dietary nitrate could effectively prevent HIRI in mice. We studied the underlying mechanism of action and found that nitrate converted to NO regulates oxidative stress by activating the NRF2 pathway. These data support the concept and the feasibility of dietary inorganic nitrate for the clinical prevention of HIRI.

## Data Availability

The raw data supporting the conclusions of this article will be made available by the authors, without undue reservation.
