# Supplementary material for: Dietary Inorganic Nitrate Protects Hepatic Ischemia-Reperfusion Injury Through NRF2-Mediated Antioxidative Stress
Source: Front Pharmacol. 2021 Jun 7;12:634115. doi: 10.3389/fphar.2021.634115 (PMC8215696; doi:10.3389/fphar.2021.634115)
Supplement: Supplementary file 1 [file DataSheet1.docx]

**Supplementary Material**

**Supplementary Table S1**

| mouse Tnf-α F | TCCCAGGTTCTCTTCAAGGGA |
| --- | --- |
| mouse Tnf-α R | GGTGAGGAGCACGTAGTCGG |
| mouse Il-1β F | CCCTGCAGCTGGAGAGTGTGGA |
| mouse Il-1β R | TGTGCTCTGCTTGTGAGGTGCTG |
| mouse Il-10 F | AGAAAAGAGAGCTCCATCATGC |
| mouse Il-10 R | TTATTGTCTTCCCGGCTGTACT |
| mouse IL-1α F | GAGAGCCGGGTGACAGTATC |
| mouse IL-1α R | TGACAAACTTCTGCCTGACG |
| mouse IL-17A F | GCTCCAGAAGGCCCTCAGACT |
| mouse IL-17A R | CCAGCTTTCCCTCCGCATTGA |
| mouse IL-27 F | CACCTCCGCTTTCAGGTGC |
| mouse IL-27 R | AGGTATAGAGCAGCTGGGGC |
| mouse Bcl-2 F | GGAAGGTAGTGTGTGTGG |
| mouse Bcl-2 R | ACTCCACTCTCTGGGTTCTTGG |
| mouse Bcl-xL F | AACATCCCAGCTTCACATAACCCC |
| mouse Bcl-xL R | GCGACCCCAGTTTACTCCATCC |
| mouse Nrf2 F | AGGACATGGAGCAAGTTTGG |
| mouse Nrf2 R | TCCTCAAAACCATGAAGGAA |
| mouse Nqo-1 F | AGGGTTCGTATTACGATCC |
| mouse Nqo-1 R | AGTACAATCAGGGCTCTTCTCG |
| mouse Ho-1 F | TCTATCGTGCTCGCATGAAC |
| mouse Ho-1 R | CTGTCTGTGAGGGACTCTGG |
| mouse Keap1 F | CAGCTACACACTAGAGGATCACA |
| mouse Keap1 R | GTGGATGCCTTCGATGGACA |
| mouse TrxR F | GCACGCGGGTTAAGGAACT |
| mouse TrxR R | TGGGCACCGTTTTCTGGTTAC |
| mouse GSTp1 F | ATGCCACCATACACCATTGTC |
| mouse GSTp1 R | GGGAGCTGCCCATACAGAC |
| mouse CAT F | CCCCTATTGCCGTTCGATTCT |
| mouse CAT R | TTCAGGTGAGTCTGTGGGTTT |
| mouse GPx1 F | GCTCACCCGCTCTTTACC |
| mouse GPx1 R | GCCGCCTTAGGAGTTGC |
| mouse Hmgb1 F | GCTGACAAGGCTCGTTATGAA |
| mouse Hmgb1 R | CCTTTGATTTTGGGGCGGTA |
| mouse S100b F | TGGTTGCCCTCATTGATGTCT |
| mouse S100b R | CCCATCCCCATCTTCGTCC |
| mouse GADPH F | GGTGAAGGTCGGTGTGAACG |
| mouse GADPH R | CTCGCTCCTGGAAGATGGTG |

**Supplementary Figure S1**


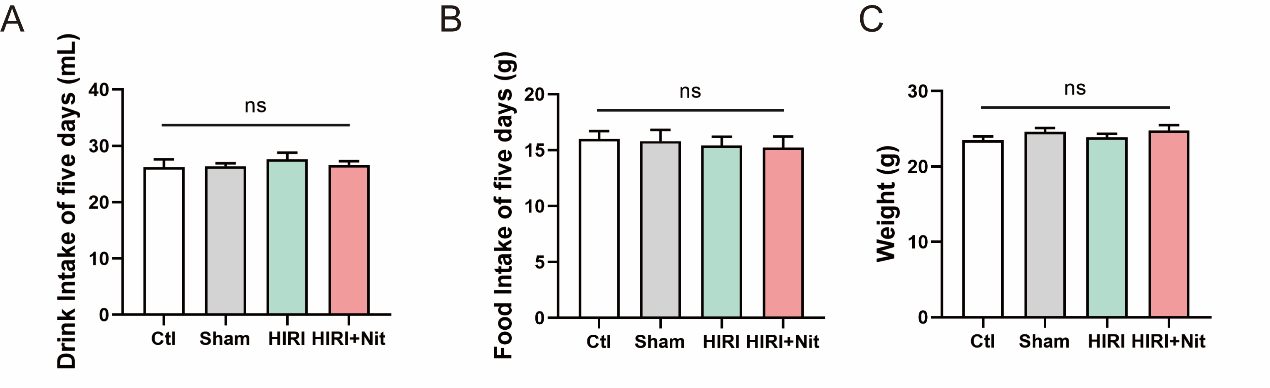


**Figure S1. After 5 days pretreatment.** (A) Drink intake, (B) food intake and (C) weight in Ctl, Sham, HIRI and HIRI+Nit groups. Data are expressed as mean ± SEM,**P*＜0.05,***P*＜0.01,****P*＜0.001, *NS* nonsignificant.
